# Increased Variability in Lower Limb Muscle Activation Is Observed with Increasing Walking Speed in Fall-Risk Older Adults

**DOI:** 10.3390/life14121551

**Published:** 2024-11-26

**Authors:** Yongnam Park, Youngsook Bae

**Affiliations:** 1 Department of Physical Therapy, Suwon Women’s University, Suwon 18333, Republic of Korea; till87@hanmail.net; 2 Department of Physical Therapy, College of Medical Science, Gachon University, 191 Hambangmoe-ro, Yeonsu-gu, Incheon 21936, Republic of Korea

**Keywords:** fall risk, muscle activation, older adults, safe walking, variability

## Abstract

This study is a cross-sectional study and aims to determine the differences in lower limb muscle activation and variability at preferred, slow, and fast walking speeds according to age and fall risk. We divided 301 participants into groups based on fall risk (fall-risk vs. non-fall-risk). We measured muscle activation and its coefficients of variation (CV) for the rectus femoris (RF), biceps femoris (BF), tibialis anterior (TA), and medial gastrocnemius muscle (GCM) at speeds 20% slower, 20% faster, and 40% faster than the preferred speed (PS). When compared by fall risk, fall-risk older adults had significantly lower GCM activity and higher CVs of RF, BF, TA, and GCM in PS. With changes in gait speed, fall-risk older adults had significantly increased CVs of RF, BF, and GCM. Our findings provide new evidence that variability rather than muscle activity increases with walking speed in older adults at risk of falls, highlighting the importance of decreasing muscle activity variability in preventing fall risk.

## 1. Introduction

In an aging society, reducing the risk of falls, which can diminish physical function, independence in daily living, and quality of life in older adults, has emerged as a major issue. The ability to walk safely is an essential physical ability required to minimize the risk of falls and maintain physical independence [1]. Gait and balance abilities are closely related and are crucial factors in predicting quality of life and mortality rates [2]. Age-related loss of muscle mass can alter muscle activity patterns and lead to decreased gait and balance abilities [3]. In particular, muscle strength and activation in the lower limbs can affect walking [4], balance ability [5,6], and fall risk [7].

Promsri et al. [8] reported that lower scores on the Short Physical Performance Battery (SPPB), which measures fall risk, are associated with increased gait instability and, consequently, a higher risk of falls. Gait instability refers to a deterioration in the consistency of gait and is represented by variability; higher gait variability indicates worse walking consistency [9], making variability a key indicator of gait consistency strongly associated with fall risk [9]. Among older adults, gait variability is more pronounced in those with greater muscle activation variability and can be closely related to walking speed [10].

Recent studies have emphasized the importance of identifying changes in lower limb muscle activity to predict and prevent falls in older adults [11]. Furthermore, older adults exhibit greater muscle activation variability than younger adults do when performing rapid repetitive movements [12]. As people age, not only can lower limb muscle strength and activation decrease, but the variability in lower limb muscle activation during walking can also increase; this is closely related to fall risk [13]. Therefore, age-related decreases in lower limb muscle activation may lead to increased muscle activation variability as walking speed increases, and this increased variability in response to changes in walking speed could be closely related to fall risk.

Previous studies have identified the muscles involved in response to changes in speed in both young and older adults [14], demonstrated a correlation between balance ability and gait variability [15], and identified specific lower limb muscles related to fall risk [16]. However, there is a relative lack of research analyzing the activation and variability of specific lower limb muscles in response to age-related changes in walking speed with an increase in age in older adults. It is particularly important to investigate changes in muscle activation and variability with changes in walking speed in older adults at risk of falls.

We hypothesized that fall-risk older adults would exhibit decreased muscle activation and greater variability in lower limb muscle activity as walking speed increases compared to non-fall-risk older adults. Therefore, we aimed to identify differences in lower limb muscle activation and variability at preferred, slow, and fast walking speeds between fall-risk and non-fall-risk older adults among community-dwelling older adults based on fall risk. Additionally, we sought to identify the patterns of muscle activation in response to changes in walking speed among older adults at risk of falls.

## 2. Materials and Methods

### 2.1. Design and Ethical Considerations

To determine the variability of muscle activity at different walking speeds, all older adults were randomly assigned to walk at different walking speeds. This cross-sectional study recruited study recruited 301 older adults between September 2023 and January 2024. Before commencing the experimental process, detailed information about the study procedure and safety was provided to the participants, who subsequently signed a written informed consent form. This study was approved by the Institutional Review Board of Gachon University (Clinical trial registration number: KCT0009118) and conducted in accordance with the Declaration that participants were assigned to different speed conditions in Helsinki (as revised in 2013).

### 2.2. Participants and Procedures

The participants were 301 community-dwelling older adults (aged 70–90 years) recruited through posters in community centers and based on telephone interviews, according to the following criteria. We included older adults (1) aged ≥70 years who were able to perform activities of daily living with or without assistive devices; (2) not currently experiencing orthopedic problems in the lower extremities or neurological disorders, such as cerebral infarction; and (3) who had not undergone surgery within 6 months due to musculoskeletal diseases of the lower limb. Individuals who had a Mini-Mental State Examination score < 24 or difficulty in walking on a treadmill and those who did not perform the measurement procedures were excluded. At the beginning of the study, 368 older adults were recruited. However, 11 older adults did not meet the inclusion criteria, and 56 were unable to perform the measurement procedures because of difficulty in walking on a treadmill.

All participants were asked to complete a questionnaire about their health status and experience of falling, and their lower-extremity strength and balance ability were measured. Subsequently, lower limb muscle activity and variability were measured using electromyography while the participants walked on a treadmill. In gait, variability is an indicator of decreased motor control, and at least 40 steps should be taken to determine variability [17]. Treadmills can be used to assess gait kinematics in physically active older adults [18]. In this study, treadmill walking was chosen to minimize measurement errors that may occur due to natural acceleration and deceleration when walking on the ground and to ensure the collection of more than 40 steps at a constant speed. Before performing treadmill walking, all participants walked 14 m to measure their preferred walking speed. To minimize errors in measuring the average preferred walking speed caused by acceleration and deceleration, the time taken to walk the middle 10 m was used after excluding the time taken to walk the initial and final 2 m. Treadmill walking generally has a slower preferred speed than overground walking [18]. Participants acclimated to treadmill walking at their PS obtained from overground walking measurements. Therefore, in this study, PS when walking on a treadmill was adjusted to ±0.1 km/h during the treadmill acclimation period. After determining the PS, the participants took a 2-min rest before walking on a treadmill. They then walked at speeds 20% slower, 20% faster, and 40% faster than their PS for 90 s each, with a rest period of approximately 1 min between trials. Muscle activity was measured for approximately 60 s, starting 20 s after the participant began walking on the treadmill.

**Figure 1:**
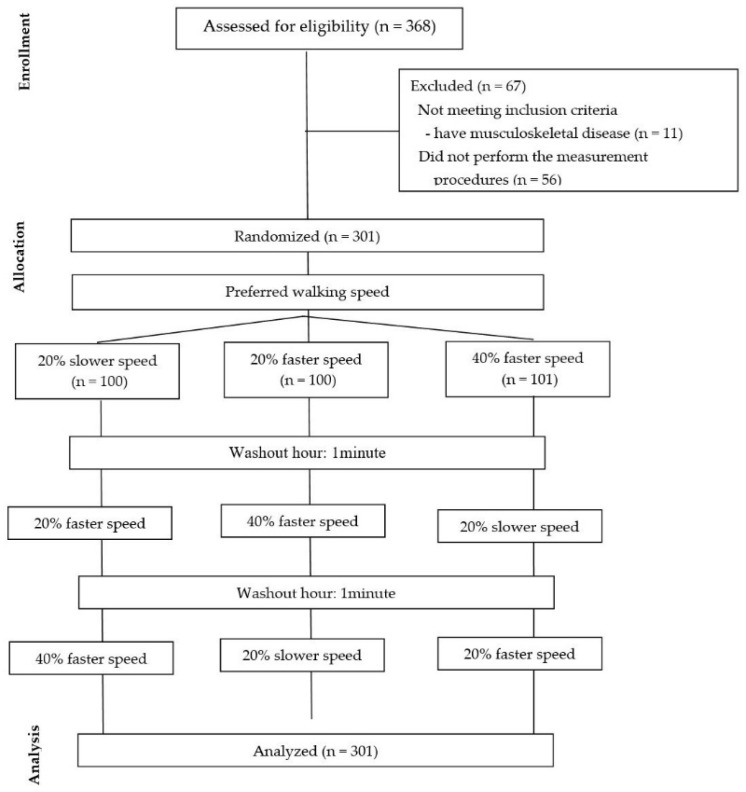
Flow of this study.

All participants had their lower limb muscle activity measured at their PS and were then randomly assigned to one of the three speed sequences (20% slower, 20% faster, 40% faster; 20–40% faster, 20% slower; and 40% faster, 20% slower, 20% faster) to have their lower limb muscle activity measured again. The order of assignment was determined using a randomization website (http://www.randomization.com (accessed on 25 September 2023)). To ensure fairness and objectivity, the randomization process was conducted by a researcher who was not directly involved with the study (Figure 1).

All data were collected from a university laboratory and a community center. The researchers were aware of the purpose of the study, but the outcome assessors were not. G*Power 3.1.9 software was used to calculate the required sample size for the study, and power = 0.8, α = 0.05, and effect size = 0.5 were determined based on a two-tailed test. The calculated sample size was 128. 

### 2.3. Data Collection

#### 2.3.1. Fall Risk

The SPPB score is a useful clinical tool for assessing fall risk for older adults that can distinguish fall risk from non-fall risk [19,20]. SPPB (0–12 points) was used to measure physical performance, and it consisted of balance, a 4-meter walk, and five-timed chair stand tests. In the balance test, the participants were asked to maintain their feet in an aligned position, then in a semi-aligned position, and finally, in a side-by-side position for 10 s each. The 4-m walk involved walking at a usual pace. For the timed chair stand test, a pre-test of five trials was conducted. The participants were asked to stand up from a chair with their arms crossed over their chests, and the time from the first sitting position to the last standing position was measured in seconds during the fifth stand. The SPPB score was calculated according to a previous study [21]. Because an SPPB score ≤ 9 is related to fall risk [20,22], in this study, participants with SPPB scores of 9 or below were classified as the fall-risk group, while those with scores of 10 or above were classified as the non-fall-risk group.

#### 2.3.2. Lower Limb Muscle Activity and Variability

Surface electromyography (sEMG) equipment (Ultium ESP, Noraxon, AZ, USA) was used to measure the activation of the lower limb muscles during walking on a treadmill. The settings for the EMG signal collection were as follows: sampling rate of 2000 Hz, band-pass filter of 10–500 Hz, and notch filter of 60 Hz. Before electrode placement, the participant’s skin was shaved and cleaned with alcohol. Disposable dipole electrodes (Ag/AgCl) were attached to the skin of the participants at the midline of the muscle belly, and electrode placement was performed according to sEMG (surface EMG for non-invasive assessment of muscles) guidelines [23]. The sEMG data were collected from four lower-extremity muscles: the rectus femoris (RF), tibialis anterior (TA), biceps femoris (BF), and gastrocnemius muscle (GCM) [24]. Using the root mean square (RMS) to measure muscle activation in older adults is effective, and the correlation between RMS values and muscle strength makes this test reliable to assess age-related muscle changes [25,26]. Therefore, in this study, to analyze differences in lower limb muscle activation at different gait speeds, sEMG signals were processed by calculating the RMS values, which are used to quantify the magnitude of EMG signals and muscle contraction intensity, and the unit is μV. The variability in the measured muscle activity was calculated as the coefficient of variation (CV), which was expressed as the ratio of the standard deviation to the mean (standard deviation/mean × 100) of each muscle activity and presented as a percentage [27].

#### 2.3.3. Statistical Analysis

SPSS 26.0 software (IBM, Armonk, NY, Watson, USA) was used for statistical analyses. Frequency and descriptive statistics were analyzed to assess the general characteristics of the participants.

Participants were stratified according to fall risk (fall-risk older adults, n = 141; non-fall-risk older adults, n = 160). A comparison of lower limb muscle activity at the preferred walking speed based on fall risk was conducted using an independent *t*-test. A one-way repeated-measures analysis of variance (ANOVA) was used to evaluate the differences in lower limb muscle activity at the PS and at the three different speeds within each group. A two-way repeated-measures ANOVA was used to examine changes between groups according to fall risk. To examine the effect of gender on lower extremity muscle activation and variability, we performed ANOVAs with gender as a covariate. The effect sizes of the interaction effect were calculated using eta-squared (η^2^), with effect sizes defined as up to 0.02 for small changes, 0.13 for moderate changes, and 0.26 for large changes. All data were expressed as mean ± standard deviation. The level of significance was set at α < 0.05.

## 3. Results

A total of 301 participants (135 men, 166 women) were included in the study. In the fall-risk classification, there were 141 older adults in the fall-risk group and 160 in the non-fall-risk group (Table 1).

**Figure 2:**
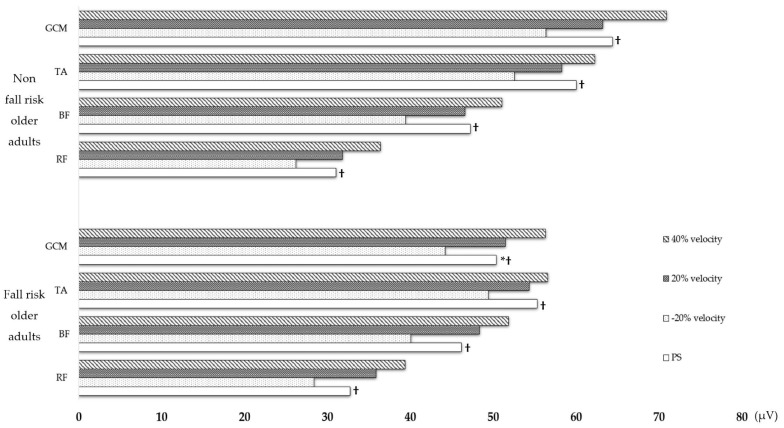
Comparison of muscle activity within and between groups in fall-risk and non-fall-risk older adults. RF: Rectus femoris, BF: Biceps femoris, TA: Tibialis anterior, GCM: Gastrocnemius medialis. * *p* < 0.05 indicates a significant difference in preferred speed between fall-risk and non-fall-risk older adults. ^†^ *p* < 0.05 indicates a significant difference within the group.

**Figure 3:**
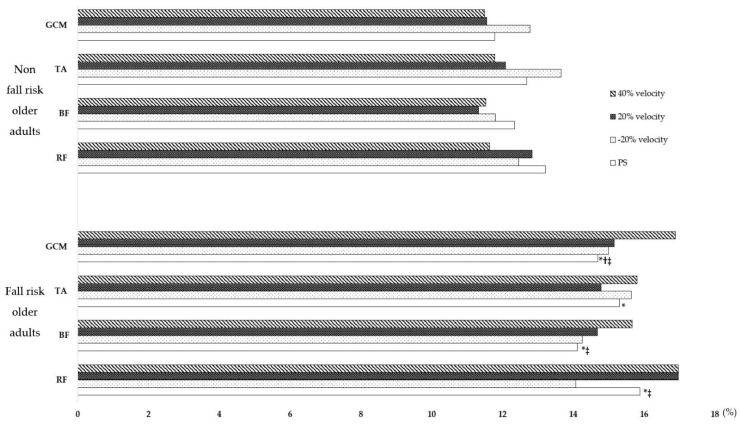
Comparison of variability within and between groups in fall-risk and non-fall-risk older adults. RF: Rectus femoris, BF: Biceps femoris, TA: Tibialis anterior, GCM: Gastrocnemius medialis. * *p* < 0.05 indicates a significant difference in preferred speed between fall-risk and non-fall-risk older adults. ^†^ *p* < 0.05 indicates a significant difference within the group. ^‡^ *p* < 0.05 indicates a significant difference between fall-risk and non-fall-risk older adults.

Comparing fall-risk and non-fall-risk older adults walking at the PS, we found that fall-risk older adults had significantly lower GCM activity and significantly higher CV for RF, BF, TA, and GCM activities than non-fall-risk older adults. Changes in muscle activity and the CV with gait speed showed significant differences in RF (*p* < 0.001, η^2^ = 0.206), BF (*p* < 0.001, η^2^ = 0.204), TA (*p* < 0.001, η^2^ = 0.155), and GCM (*p* < 0.001, η^2^ = 0.157) activities in fall-risk older adults compared with non-fall-risk older adults. Compared to the PS, in the fall risk group, muscle activity of RF (*p* < 0.001, η^2^ = 0.206), BF (*p* < 0.001, η^2^ = 0.204), TA (*p* < 0.001, η^2^ = 0.155), and GCM (*p* < 0.001, η^2^ = 0.157) significantly decreased as the speed was 20% slower, and muscle activity significantly increased as the speed was 40% faster in the fall risk group. The CV for RF (*p* = 0.002, η^2^ = 0.033) and GCM activities (*p* < 0.003, η^2^ = 0.033) were also significantly reduced at a 20% slower speed and significantly increased at 40% faster speed. In non-fall-risk older adults, there were significant differences in RF (*p* < 0.001, η^2^ = 0.232), BF (*p* < 0.001, η^2^ = 0.239), TA (*p* < 0.001, η^2^ = 0.209), and GCM (*p* < 0.001, η^2^ = 0.298) activities, with a significant decrease in muscle activity at 20% slower speed and a significant increase in muscle activity at 40% faster speed. There was also a significant difference in the CV for TA (*p* < 0.001, η^2^ = 0.044) and GCM (*p* = 0.004, η^2^ = 0.027) activities, with a significant decrease in the CV as speed increased. The CV for RF activity was also significantly reduced at a 40% faster speed; however, it was not significantly different in non-fall-risk older adults. Comparisons between the two groups showed that fall-risk older adults had significantly greater increases in the CV for RF (*p* = 0.002, η^2^ = 0.016), BF (*p* = 0.047, η^2^ = 0.009, and GCM (*p* < 0.001, η^2^ = 0.022) activities at 40% faster speed, compared with non-fall-risk older adults. Moreover, in fall-risk and non-fall-risk older adults, gender did not affect lower extremity muscle activation or variability (Table 2 and Table 3) (Figure 2 and Figure 3).

## 4. Discussion

We compared lower limb muscle activity and variability according to walking speed between fall-risk and non-fall-risk groups to identify muscle activation in response to changes in speed and to quantitatively analyze the specific effects of increased walking speed on muscle activity variability. Fall-risk older adults had lower GCM activation at their preferred speed compared to non-fall-risk older adults, with increased CV for RF, BF (hamstrings), TA, and GCM activities. Furthermore, as walking speed increased, the CV in non-fall-risk older adults decreased, while it increased in fall-risk older adults, with significant differences, particularly in the CV for RF and GCM activities.

In fall-risk older adults, the activation of the RF, BF, and GCM increased significantly at speed 40% faster than the PS, similar to that in non-fall-risk older adults. However, the TA was not significantly activated in fall-risk older adults. Lim et al. [28] found that stroke patients with reduced balance showed greater cortical input to the RF than to the TA on the paretic side during walking, which increased gait variability. This indicates that inadequate activation of the TA with an increase in walking speed can lead to decreased balance or an increased risk of falls. Moreover, older adults at risk of falls had decreased GCM activation as walking speed increased, compared with older adults who were not at risk of falls. These results suggest that the increased RF muscle activation with walking speed represents a compensatory mechanism for weakened GCM muscles during walking, whereas the reduced GCM muscle activation is associated with higher fall risk [29,30]. Therefore, older adults with decreased balance ability may exhibit inefficient lower limb muscle activation patterns to maintain walking stability as walking speed increases, which may affect their ability to walk, particularly at higher speeds [10].

Walking variability is a sensitive indicator of abnormal walking [31]. Aging affects muscle strength and force control, increases force output variability, and decreases motor performance in older adults [32]. Older adults with higher variability in lower-extremity muscle activation have greater gait variability at different walking speeds, which increases the risk of falls [10]. Our results showed that fall-risk older adults had higher CVs for RF, BF, TA, and GCM activities at their PS than non-fall-risk older adults. In addition, in non-fall-risk adults, the CV decreased when walking speed increased, whereas fall-risk older adults had significantly higher CVs for RF and GCM activities.

Recently, a study [33] reported a significant correlation between muscle activation variability measured by EMG and task performance and variability in RF activity increases during walking with age [34]. This suggests that increased muscle activation variability during repetitive movements may lead to difficulty in task performance and that increased variability in RF activity in older adults may lead to walking difficulties. Lim et al. [28] also found that, in patients with impaired balance ability, the RF had greater activation than the TA, which led to increased gait variability. Therefore, variability in RF activity is more important than TA activation in maintaining a stable gait.

Additionally, our results showed that variability increased in fall-risk older adults compared to non-fall-risk older adults when walking 20% slower and 40% faster than their preferred speed (PS). This finding is consistent with previous studies that showed muscle activity variability increases as walking speed decreases [35]. Therefore, additional caution is needed when training at slower walking speeds [36]. Furthermore, when walking speed increased by 40% above PS, the CV decreased in non-fall-risk older adults, whereas it increased in fall-risk older adults, with significant differences, particularly in RF and GCM activities. Reducing variability may help achieve stable walking and reduce the risk of falls [37]. These results suggest that muscle activity stability decreases as walking speed increases, indicating that the muscle control mechanisms in older adults function differently depending on their fall-risk status. This implies that abnormal walking and fall risk can be identified not only through spatiotemporal variability but also through variability in RF and GCM muscle activity. Therefore, identifying changes in variability at 40% faster than PS may be a key factor in detecting balance issues and fall risk in older adults.

This study has some limitations. First, only older adults who were able to walk on a treadmill in a laboratory setting were included. This may not fully reflect walking in daily life and may have potential differences from real walking. Second, some older adults were unable to perform the study procedures because of fear of walking on a treadmill, and muscle activation may be affected by psychological factors such as fear [38,39]. Third, muscle activity was assessed using sEMG; however, direct measurements of muscle strength were not performed, and muscle coordination was not evaluated through time-dependent EMG analysis, which could have provided insights into muscle coordination during gait. Additionally, this study did not consider that a 40% increase in walking speed might be excessive for some older adults.

Despite these limitations, this is the first study to systematically analyze muscle activity and variability according to changes in walking speed based on fall risk. We identified the characteristics of muscle activation during walking in older adults at risk of falls and provided foundational data for fall prevention. By further understanding the mechanism of the muscle response according to changes in walking speed, it will be possible to improve walking safety in older adults. Future research should include analysis of walking in real-life environments and verification of the effects of specific rehabilitation programs aimed at improving lower limb muscle activation and variability.

## 5. Conclusions

This study identified lower limb muscle activation and variability during walking according to fall risk and found that in older adults at risk for falls, muscle activation variability increased with changes in walking speed. In particular, in fall-risk older adults, variability, rather than muscle activation itself, increased more significantly as walking speed increased. These findings suggest that muscle activation variability, rather than muscle activation itself, is more closely related to fall risk. Therefore, reducing muscle activation variability may be an important strategy for fall prevention in at-risk older adults.

## Figures and Tables

**Table 1:** General characteristics of subjects according to age and fall risk level.

	Fall-Risk Older Adults (n = 141)	Non-Fall-Risk Older Adults (n = 160)	t	*p*
Sex (male/female)	47/94	72/88		
Age (years)	79.97 ± 5.69	78.81 ± 5.13	1.857	0.064
Weight (kg)	59.27 ± 9.51	61.23 ± 10.08	−1.726	0.085
Height (cm)	155.62 ± 8.05	159.33 ± 8.14	−3.956	<0.001
SPPB (score)	7.74 ± 1.87	11.47 ± 0.70	−23.326	<0.001

SPPB: Short physical performance battery.

**Table 2:** Comparison of muscle activity and CV between fall5risk older and non-fall-risk older adults.

	Fall Risk Older Adults				Non-Fall Risk Older Adults			
	PS	−20% Speed	20% Speed	40% Speed	PS	−20% Speed	20% Speed	40% Speed
RF activity (μV)	32.69 ± 16.13	28.38 ±15.23 ^b^	35.85 ± 19.78 ^b^	39.38 ± 23.82 ^b^	31.02 ± 15.85	26.16 ± 13.88 ^b^	31.81 ± 16.05	36.38 ± 17.93 ^b^
RF CV (%)	15.88 ± 10.28 ^a^	14.07 ± 7.39 ^b^	16.97 ± 9.42	16.86 ± 9.52	13.22 ± 6.66	12.45 ± 5.85	12.84 ± 7.02	11.64 ± 5.29 ^b^
BF activity (μV)	46.15 ± 21.97	40.04 ± 19.96 ^b^	48.35 ± 24.24 ^b^	51.82 ± 24.83 ^b^	47.20 ± 21.14	39.40 ± 19.42 ^b^	46.55 ± 21.72	51.02 ± 23.76 ^b^
BF CV (%)	14.12 ± 6.18 ^a^	14.25 ± 7.16	14.68 ± 8.01	15.66 ± 10.09 ^b^	12.34 ± 8.72	11.80 ± 4.30	11.32 ± 4.16	11.53 ± 6.42
TA activity (μV)	55.27 ± 23.96	49.37 ± 22.57 ^b^	54.34 ± 24.52	56.57 ± 23.29	60.00 ± 24.67	52.54 ± 22.00 ^b^	58.24 ± 23.04 ^b^	62.24 ± 23.66 ^b^
TA CV (%)	15.31 ± 8.81 ^a^	15.64 ± 7.72	14.78 ± 7.35	15.80 ± 7.53	12.68 ± 4.79	13.66 ± 5.94 ^b^	12.09 ± 4.71	11.78 ± 4.41 ^b^
GCM activity (μV)	50.36 ± 20.96 ^a^	44.17 ± 20.92 ^b^	51.45 ± 20.78	56.31 ± 25.67 ^b^	64.34 ± 25.61	56.33 ± 26.04 ^b^	63.18 ± 26.00	70.90 ± 27.77 ^b^
GCM CV (%)	14.69 ± 6.30 ^a^	14.99 ± 6.46	15.15 ± 6.00	16.88 ± 7.73 ^b^	11.78 ± 4.07	12.78 ± 5.18	11.56 ± 4.42	11.50 ± 4.43

PS: preferred speed, RF: Rectus femoris, BF: Biceps femoris, TA: Tibialis anterior, GCM: Gastrocnemius medialis, CV: coefficient of variation. ^a^ *p*< 0.05 statistical analysis for PS comparison between two groups using independent *t*-test. ^b^ *p* < 0.05, a significant difference compared to PS using one-way repeated ANOVA.

**Table 3:** Statistical analysis of muscle activity and variability within and between groups in fall-risk and non-fall-risk older adults.

	Fall-Risk Older Adults ^a^				Non-Fall-Risk Older Adults ^a^				Between Groups ^c^			
	F	*p*	η^2^	adj ^b^	F	*p*	η^2^	adj ^b^	F	*p*	η^2^	adj ^b^
RF (μV)	36.504	<0.001	0.206	0.912	48.029	<0.001	0.232	0.200	1.108	0.365	0.004	0.510
RF CV (%)	4.867	0.340	0.008	0.455	2.712	0.0.437	0.006	0.694	4.876	0.003	0.015	0.311
BF (μV)	36.026	<0.001	0.204	0.495	49.823	<0.001	0.239	0.413	1.218	0.302	0.004	0.175
BF CV (%)	2.137	0.095	0.015	0.461	1.086	0.355	0.007	0.413	2.664	0.029	0.010	0.415
TA (μV)	25.850	<0.001	0.155	0.100	41.947	<0.001	0.209	0.488	1.605	0.187	0.005	0.159
TA CV (%)	0.866	0.459	0.006	0.897	7.335	<0.001	0.044	0.310	2.321	0.074	0.008	0.957
GCM (μV)	26.356	<0.001	0.157	0.662	67.642	<0.001	0.298	0.135	1.334	0.262	0.004	0.195
GCM CV (%)	4.849	0.003	0.033	0.280	4.438	0.004	0.027	0.179	6.624	<0.001	0.022	0.058

RF: Rectus femoris, BF: Biceps femoris, TA: Tibialis anterior, GCM: Gastrocnemius medialis, CV: coefficient of variation. ^a^ Statistical analysis was performed using one-way repeated-measures ANOVA. ^b^ Adjusted *p*-value, statistical analysis was performed by adjusting gender as a covariate. ^c^ Statistical analysis was performed using the two-way repeated-measures ANOVA.

## Data Availability

Data presented in this study are available from the corresponding author upon request (Data cannot be made public due to privacy or ethical restrictions).

## References

[1] Callisaya M.L., Blizzard L., Schmidt M.D., McGinley J.L., Srikanth V.K. (2010). Ageing and gait variability—A population-based study of older people. Age Ageing.

[2] Ellis T., Cavanaugh J.T., Earhart G.M., Ford M.P., Foreman K.B., Dibble L.E. (2011). Which measures of physical function and motor impairment best predict quality of life in Parkinson’s disease?. Park. Relat. Disord..

[3] Distefano G., Goodpaster B.H. (2018). Effects of exercise and aging on skeletal muscle. Cold Spring Harb. Perspect. Med..

[4] Stotz A., Hamacher D., Zech A. (2023). Relationship between muscle strength and gait parameters in healthy older women and men. Int. J. Environ. Res. Public Health.

[5] Ishigaki E.Y., Ramos L.G., Carvalho E.S., Lunardi A.C. (2014). Effectiveness of muscle strengthening and description of protocols for preventing falls in the elderly: A systematic review. Braz. J. Phys. Ther..

[6] Wu R., Zhang Y., Bai J.-J., Sun J., Bao Z.-J., Wang Z. (2020). Impact of lower limb muscle strength on walking function beyond aging and diabetes. J. Int. Med. Res..

[7] Bobowik P., Wiszomirska I. (2020). Diagnostic dependence of muscle strength measurements and the risk of falls in the elderly. Int. J. Rehabil. Res..

[8] Promsri A., Cholamjiak P., Federolf P. (2023). Walking stability and risk of falls. Bioengineering.

[9] Hausdorff J.M., Rios D.A., Edelberg H.K. (2001). Gait variability and fall risk in community-living older adults: A 1-year prospective study. Arch. Phys. Med. Rehabil..

[10] LaRoche D., Joshi P. (2023). Muscle Activation Variability as a Determinant of Gait Variability in Older Adults. Innov. Aging.

[11] Afschrift M., van Deursen R., De Groote F., Jonkers I. (2019). Increased use of stepping strategy in response to medio-lateral perturbations in the elderly relates to altered reactive tibialis anterior activity. Gait Posture.

[12] Christou E.A., Carlton L.G. (2001). Old adults exhibit greater motor output variability than young adults only during rapid discrete isometric contractions. J. Gerontol. Ser. A Biol. Sci. Med. Sci..

[13] Shin S., Valentine R.J., Evans E.M., Sosnoff J.J. (2012). Lower extremity muscle quality and gait variability in older adults. Age Ageing.

[14] Schmitz A., Silder A., Heiderscheit B., Mahoney J., Thelen D.G. (2009). Differences in lower-extremity muscular activation during walking between healthy older and young adults. J. Electromyogr. Kinesiol..

[15] Tan K., Li X., Li R. Correlation analysis of gait variability and center of pressure during stance in healthy older adults. Proceedings of the 2022 International Conference on Wearables, Sports and Lifestyle Management (WSLM), IEEE.

[16] Mehmood A., Nadeem A., Ashraf M., Siddiqui M.S., Rizwan K., Ahsan K. (2020). A fall risk assessment mechanism for elderly people through muscle fatigue analysis on data from body area sensor network. IEEE Sens. J..

[17] Rennie L., Löfgren N., Moe-Nilssen R., Opheim A., Dietrichs E., Franzén E. (2018). The reliability of gait variability measures for individuals with Parkinson’s disease and healthy older adults–The effect of gait speed. Gait Posture.

[18] Malatesta D., Canepa M., Menendez Fernandez A. (2017). The effect of treadmill and overground walking on preferred walking speed and gait kinematics in healthy, physically active older adults. Eur. J. Appl. Physiol..

[19] Lauretani F., Ticinesi A., Gionti L., Prati B., Nouvenne A., Tana C., Meschi T., Maggio M. (2019). Short-Physical Performance Battery (SPPB) score is associated with falls in older outpatients. Aging Clin. Exp. Res..

[20] Welch S.A., Ward R.E., Beauchamp M.K., Leveille S.G., Travison T., Bean J.F. (2021). The Short Physical Performance Battery (SPPB): A quick and useful tool for fall risk stratification among older primary care patients. J. Am. Med. Dir. Assoc..

[21] Guralnik J.M., Simonsick E.M., Ferrucci L., Glynn R.J., Berkman L.F., Blazer D.G., Scherr P.A., Wallace R.B. (1994). A short physical performance battery assessing lower extremity function: Association with self-reported disability and prediction of mortality and nursing home admission. J. Gerontol..

[22] Veronese N., Bolzetta F., Toffanello E.D., Zambon S., De Rui M., Perissinotto E., Coin A., Corti M.-C., Baggio G., Crepaldi G. (2014). Association between short physical performance battery and falls in older people: The Progetto Veneto Anziani study. Rejuvenation Res..

[23] Hermens H.J., Freriks B., Disselhorst-Klug C., Rau G. (2000). Development of recommendations for SEMG sensors and sensor placement procedures. J. Electromyogr. Kinesiol..

[24] Valencia O., Cristi I., Ahumada D., Meza K., Salas R., Weinstein A., Guzmán-Venegas R. (2020). The initial impact with forefoot increases the muscular activity of gastrocnemius during running. A quantitative study of electromyographic activity. Retos.

[25] Wang W., Li K., Wei N., Yue S., Yin C. Reaching forward test during standing: A comparison between the young and the elderly: A comparison between the young and the elderly. Proceedings of the 2017 Chinese Automation Congress (CAC), IEEE.

[26] Watanabe K., Kouzaki M., Ogawa M., Akima H., Moritani T. (2018). Relationships between muscle strength and multi-channel surface EMG parameters in eighty-eight elderly. Eur. Rev. Aging Phys. Act..

[27] Moon Y., Sung J., An R., Hernandez M.E., Sosnoff J.J. (2016). Gait variability in people with neurological disorders: A systematic review and meta-analysis. Hum. Mov. Sci..

[28] Lim H., Cleland B., Madhavan S. (2023). Functional connectivity of proximal and distal lower limb muscles and impact on gait variability in stroke. Gait Posture.

[29] Kim N., Park J., Shin H., Bae Y. (2022). Gastrocnemius medial head stiffness is associated with potential fall risk in community-dwelling older adults. Healthcare.

[30] Kirkwood R.N., Trede R.G., de Souza Moreira B., Kirkwood S.A., Pereira L.S.M. (2011). Decreased gastrocnemius temporal muscle activation during gait in elderly women with history of recurrent falls. Gait Posture.

[31] Wang Y., Mukaino M., Ohtsuka K., Otaka Y., Tanikawa H., Matsuda F., Tsuchiyama K., Yamada J., Saitoh E. (2020). Gait characteristics of post-stroke hemiparetic patients with different walking speeds. Int. J. Rehabil. Res..

[32] Vaillancourt D.E., Larsson L., Newell K.M. (2003). Effects of aging on force variability, single motor unit discharge patterns, and the structure of 10, 20, and 40 Hz EMG activity. Neurobiol. Aging.

[33] Aoyama T., Kohno Y. (2020). Temporal and quantitative variability in muscle electrical activity decreases as dexterous hand motor skills are learned. PLoS ONE.

[34] Bailey C.A., Porta M., Pilloni G., Arippa F., Pau M., Côté J.N. (2019). Sex-independent and dependent effects of older age on cycle-to-cycle variability of muscle activation during gait. Exp. Gerontol..

[35] Dzeladini F., Grappe A., Simpson C., Wu A., Ijspeert A. Muscle activation variability is inversely correlated with walking speed. Proceedings of the Converging Clinical and Engineering Research on Neurorehabilitation II: 3rd International Conference on NeuroRehabilitation (ICNR 2016).

[36] Escalona M.J., Bourbonnais D., Goyette M., Le Flem D., Duclos C., Gagnon D.H. (2021). Effects of varying Overground walking speeds on lower-extremity muscle synergies in healthy individuals. Mot. Control.

[37] Ronthal M. (2019). Gait disorders and falls in the elderly. Med. Clin..

[38] Ammendolia C., Schneider M., Williams K., Zickmund S., Hamm M., Stuber K., Tomkins-Lane C., Rampersaud Y.R. (2017). The physical and psychological impact of neurogenic claudication: The patients’ perspectives. J. Can. Chiropr. Assoc..

[39] Mullen S.P., McAuley E., Satariano W.A., Kealey M., Prohaska T.R. (2012). Physical activity and functional limitations in older adults: The influence of self-efficacy and functional performance. J. Gerontol. Ser. B Psychol. Sci. Soc. Sci..

